# Impact of Operator Specialization and On‐Site Evaluation on Nondiagnostic Thyroid Fine‐Needle Aspiration: A Comparative Study

**DOI:** 10.1002/dc.70161

**Published:** 2026-06-16

**Authors:** Fatlinda Sadiku, Marija Škerlj, Vesna Ramljak

**Affiliations:** ^1^ Department of Histology and Embryology, Faculty of Medicine University of Prishtina Prishtina Kosovo; ^2^ Institute of Pathology University Clinical Center of Kosovo Prishtina Kosovo; ^3^ Department of Clinical Cytology, Hospital for Tumors University Hospital Center “Sisters of Charity” Zagreb Croatia; ^4^ Department of Pathology, School of Medicine University of Zagreb Zagreb Croatia

**Keywords:** bethesda system, fine‐needle aspiration cytology, ROSE, specimen adequacy, thyroid FNA

## Abstract

**Background:**

Thyroid fine‐needle aspiration (FNA) is the primary diagnostic method for evaluating thyroid nodules; however, nondiagnostic results remain a significant limitation, often leading to repeat procedures and delayed diagnosis. Operator specialization and rapid on‐site evaluation (ROSE) have been proposed as strategies to improve specimen adequacy.

**Methods:**

This retrospective comparative study included all thyroid FNA procedures performed in 2025 at the University Clinical Center of Kosovo (UCCK), where procedures were performed by endocrinologists and radiologists without routine rapid on‐site evaluation (ROSE), and at the Hospital for Tumors, UHC “Sisters of Charity”, Zagreb, where procedures were performed by interventional cytopathologists with routine ROSE implementation. Cytology results were classified according to The Bethesda System for Reporting Thyroid Cytopathology. The proportion of nondiagnostic results between institutions was compared using the chi‐square test.

**Results:**

A total of 2171 thyroid FNA procedures were analyzed (1728 at UCCK and 443 at Hospital for Tumors). The nondiagnostic rate was significantly higher at UCCK (182/1728; 10.5%) compared with Hospital for Tumors (11/443; 2.5%) (*p* < 0.0001), corresponding to an odds ratio of 4.62 (95% CI, 2.50–8.54). Repeat FNA was performed in 36.3% of nondiagnostic cases at UCCK, of which 81.8% yielded a diagnostic Bethesda category.

**Conclusion:**

A significantly lower rate of nondiagnostic thyroid FNA results was observed in the center with interventional cytologists and routine ROSE implementation. These findings support the role of operator specialization and on‐site adequacy assessment in improving specimen adequacy and reducing the need for repeat procedures.

## 1 Introduction

Thyroid nodules are a common clinical problem, and fine‐needle aspiration cytology (FNAC) is the primary diagnostic modality for their initial evaluation due to its safety, cost‐effectiveness, and high diagnostic accuracy when adequate material is obtained [1]. Despite advances in ultrasound‐guided techniques, nondiagnostic (unsatisfactory) FNAC specimens remain a significant limitation in thyroid cytopathology and may lead to repeat procedures and delays in diagnosis [2, 3]. Multiple factors influence specimen adequacy, including nodule characteristics, sampling technique, and operator experience [2, 4, 5, 6, 7].

The Bethesda System for Reporting Thyroid Cytopathology (TBSRTC) provides a standardized framework for reporting FNA results and estimating the risk of malignancy [8]. Importantly, Bethesda Category I is not clinically insignificant, with reported malignancy rates ranging from approximately 5% to 20%, particularly in selected or repeatedly nondiagnostic cases [9, 10]. Therefore, minimizing nondiagnostic results is an important quality goal in thyroid FNA practice [11].

Operator‐related factors play a central role in specimen adequacy. Previous studies have demonstrated that diagnostic performance varies according to operator experience and procedural volume [12, 13]. Rapid on‐site evaluation (ROSE) has been proposed as a strategy to improve adequacy by allowing immediate assessment of specimen quality and additional sampling when necessary, and has been associated with improved diagnostic yield in several studies [14, 15, 16]. However, variability in nondiagnostic rates between institutions persists, reflecting differences in procedural practices and implementation of quality measures [17, 18].

In this context, the present study aimed to compare the rate of nondiagnostic thyroid FNA results between two tertiary centers with different procedural approaches, with particular emphasis on operator profile and the use of ROSE. We hypothesized that differences in operator specialization and availability of on‐site adequacy assessment contribute to variability in nondiagnostic rates and may impact the need for repeat procedures.

## 2 Methods

### 2.1 Study Design and Setting

This retrospective, observational, comparative study was conducted at two tertiary institutions: the Institute of Pathology, University Clinical Center of Kosovo (UCCK), Prishtina—Kosovo, and the Department of Clinical Cytology, Hospital for Tumors, UHC “Sisters of Charity”, Zagreb—Croatia.

### 2.2 Case Selection

All thyroid fine‐needle aspiration (FNA) procedures performed between January 1 and December 31, 2025, were included in the primary analysis. Only cases reported according to The Bethesda System for Reporting Thyroid Cytopathology (TBSRTC) were analyzed. Cases with incomplete documentation or without a final cytological diagnosis were excluded.

Repeat aspirations of the same lesion were not excluded, as the primary analysis was procedure‐based and aimed to assess the proportion of nondiagnostic results per FNA procedure.

### 2.3 Procedural Characteristics and Cytologic Evaluation

At the Hospital for Tumors, UHC “Sisters of Charity”, thyroid FNA procedures were performed by interventional cytopathologists with routine implementation of ROSE. A standardized single‐pass technique was routinely used with immediate adequacy assessment. At UCCK, thyroid FNA procedures were performed by endocrinologists and radiologists, and ROSE was not routinely available. Due to the retrospective nature of the study and the involvement of multiple departments, the number of needle passes per procedure was not consistently documented at UCCK.

Nondiagnostic (unsatisfactory) thyroid FNA samples were defined according to the criteria of The Bethesda System for Reporting Thyroid Cytopathology (TBSRTC). Specimens were considered nondiagnostic if they lacked adequate follicular epithelial cellularity, defined as fewer than six groups of well‐visualized follicular cells, each group containing at least 10 cells, or if the samples were compromised by poor preservation, obscuring blood, or consisted predominantly of cyst fluid without sufficient epithelial components. These diagnostic criteria were applied consistently at both institutions.

At both institutions, cytological specimens were prepared using conventional smear techniques. Air‐dried smears stained with May–Grünwald–Giemsa (MGG) were used for ROSE and routine cytologic evaluation. Liquid‐based cytology and cell block preparations were not routinely performed.

All cytology reports were classified according to TBSRTC. In addition to the analysis of Bethesda Category I rates, the distribution of all Bethesda categories (I–VI) was compared between institutions.

### 2.4 Data Collection

Data were retrieved from institutional laboratory archives and included:

- Total number of thyroid FNA procedures.
- Distribution of Bethesda categories.
- Number of procedures categorized as Bethesda Category I (nondiagnostic/unsatisfactory)
- Operator profile and procedural characteristics.
- Repeat FNA outcomes following an initial Bethesda Category I diagnosis.

All data were anonymized prior to analysis.

### 2.5 Statistical Analysis

Descriptive statistics were used to calculate frequencies and percentages. The proportion of Bethesda Category I procedures between institutions was compared using the Chi‐square test. Odds ratios (ORs) with 95% confidence intervals (CIs) were calculated. A *p* < 0.05 was considered statistically significant.

## 3 Results

During the study period (January 1–December 31, 2025), a total of 2171 thyroid fine‐needle aspiration (FNA) procedures were analyzed, including 1728 procedures performed at University Clinical Center of Kosovo (UCCK) and 443 procedures at Department of Clinical Cytology, Hospital for Tumors, UHC “Sisters of Charity”.

At UCCK, 182 of 1728 procedures (10.5%; 95% CI, 9.2%–12.1%) were categorized as Bethesda Category I (nondiagnostic/unsatisfactory). In contrast, at Hospital for Tumors, UHC “Sisters of Charity”, 11 of 443 procedures (2.5%; 95% CI, 1.4%–4.4%) were classified as Bethesda Category I. The difference in nondiagnostic rates between institutions was statistically significant (Chi‐square test, *p* < 0.0001). The odds of obtaining a nondiagnostic result were significantly higher at UCCK (OR 4.62; 95% CI, 2.50–8.54).

The overall distribution of Bethesda categories also differed between institutions. At UCCK, Bethesda Category II accounted for 1303 cases (75.4%), Bethesda Category III for 159 cases (9.2%), Bethesda Category IV for 43 cases (2.5%), Bethesda Category V for 32 cases (1.9%), and Bethesda Category VI for 9 cases (0.5%). At Hospital for Tumors, UHC “Sisters of Charity”, 325 cases (73.4%) were classified as Bethesda Category II, 58 cases (13.1%) as Category III, 21 cases (4.7%) as Category IV, and 14 cases each (3.2%) as Categories V and VI. A detailed comparison of Bethesda category distribution between institutions is presented in Table 1.

**TABLE 1:** Distribution of bethesda categories between institutions (2025).

Bethesda category	UCCK *n* (%)	Hospital for tumors, UHC “Sisters of charity” *n* (%)
I (Nondiagnostic)	182 (10.5)	11 (2.5)
II (Benign)	1303 (75.4)	325 (73.4)
III (AUS)	159 (9.2)	58 (13.1)
IV (Follicular neoplasm)	43 (2.5)	21 (4.7)
V (Suspicious for malignancy)	32 (1.9)	14 (3.2)
VI (Malignant)	9 (0.5)	14 (3.2)
Total	1728 (100)	443 (100)

### 3.1 Secondary Analysis: Repeat FNA Following Initial Bethesda Category I

At UCCK, repeat FNA within the 2025 study period was documented in 66 of 182 Bethesda Category I cases (36.3%). On repeat aspiration, 47 cases (71.2%) were reclassified as Bethesda Category II, 6 cases (9.1%) as Bethesda Category III, and 1 case (1.5%) as Bethesda Category IV. Twelve cases (18.2%) remained Bethesda Category I. Overall, 54 of 66 repeat procedures (81.8%) yielded a diagnostic Bethesda category (II–IV).

At Hospital for Tumors, UHC “Sisters of Charity”, repeat FNA within the study period was documented in 3 of 11 Bethesda Category I cases (27.3%). Of these, 1 case (33.3%) was reclassified as Bethesda Category II, while 2 cases (66.7%) remained nondiagnostic.

The distribution of repeat FNA outcomes for both institutions is summarized in Table 2.

**TABLE 2:** Outcomes of repeat fine‐needle aspiration following initial bethesda category I.

Repeat FNA outcome	UCCK *n* (%)	Hospital for tumors, UHC “Sisters of Charity” *n* (%)
Total bethesda I cases	182	11
Repeat FNA performed	66 (36.3)	3 (27.3)
Bethesda II on repeat FNA	47 (71.2)	1 (33.3)
Bethesda III on repeat FNA	6 (9.1)	0
Bethesda IV on repeat FNA	1 (1.5)	0
Remained bethesda I	12 (18.2)	2 (66.7)
Diagnostic conversion rate*	54/66 (81.8)	1/3 (33.3)

* Diagnostic conversion rate defined as the proportion of repeat FNAs yielding a diagnostic bethesda category (II–IV).

## 4 Discussion

In this retrospective comparative study, we observed a significant difference in the rate of Bethesda Category I (nondiagnostic) thyroid FNA results between two tertiary institutions. The proportion of unsatisfactory procedures was 10.5% at the Institute of Pathology, University Clinical Center of Kosovo (UCCK), compared with 2.5% at Hospital for Tumors, UHC “Sisters of Charity”. This difference was statistically significant, with an approximately 4.6‐fold higher odds of nondiagnostic results at UCCK.

According to The Bethesda System for Reporting Thyroid Cytopathology (TBSRTC), nondiagnostic rates should ideally remain below 10% in experienced centers [1, 8]. While the rate observed at Hospital for Tumors, UHC “Sisters of Charity” falls well within the recommended range, the rate at UCCK slightly exceeds this threshold. Variability in nondiagnostic rates across institutions has been reported in several studies and may reflect differences in operator experience, procedural technique, case volume, and quality assurance practices [7, 17, 18].

Several differences between the two institutions, including operator background, procedural volume, and the availability of ROSE, may have contributed to the observed variation in nondiagnostic rates. Due to the retrospective and comparative design of the study, it is not possible to determine the relative contribution of each individual factor. Therefore, the findings should be interpreted as reflecting an association between institutional practices and specimen adequacy rather than a direct causal relationship.

One of the major differences between the two centers in our study was the operator profile. At Hospital for Tumors, UHC “Sisters of Charity”, all FNAs were performed by interventional cytologists, whereas at UCCK procedures were performed by endocrinologists and radiologists. Previous studies have demonstrated that operator experience and specialization significantly influence specimen adequacy and diagnostic yield [6, 12, 13]. Dedicated cytology‐performed FNAs may allow better targeting of the lesion, optimized sampling technique, and improved slide preparation, potentially contributing to lower nondiagnostic rates.

Another important procedural distinction was the routine implementation of ROSE at Hospital for Tumors, UHC “Sisters of Charity”. ROSE allows immediate assessment of specimen adequacy and enables additional needle passes during the same procedure when the initial sample is insufficient [14]. Several institutional studies have demonstrated that ROSE significantly reduces nondiagnostic rates and improves diagnostic performance [4, 5, 16]. Furthermore, a systematic review and meta‐analysis confirmed that ROSE is associated with a statistically significant improvement in specimen adequacy in thyroid FNA [15].

Interestingly, the distribution of Bethesda categories also differed between institutions, particularly regarding Bethesda Category III. A relatively higher proportion of Bethesda Category III cases was observed at Hospital for Tumors, UHC “Sisters of Charity” compared with UCCK. This finding may reflect differences in diagnostic thresholds and interpretative practices between institutions, particularly in the use of indeterminate categories. Therefore, variation in nondiagnostic rates may not be attributable solely to procedural factors, but may also partially reflect differences in cytopathologic classification practices.

Importantly, Bethesda Category I should not be regarded as a clinically trivial classification. Large multicenter studies and institutional analyses have demonstrated that the risk of malignancy in this category ranges from approximately 5% to nearly 20%, particularly in surgically excised or repeatedly nondiagnostic nodules [9, 10, 17]. Therefore, higher nondiagnostic rates may have relevant clinical consequences, including repeat procedures, delayed diagnosis, increased patient anxiety, and additional healthcare utilization.

Our secondary analysis provides additional insight into the clinical implications of nondiagnostic results. At UCCK, repeat FNA was performed in 36.3% of Bethesda Category I cases during the study period. Importantly, 81.8% of these repeat procedures yielded a diagnostic Bethesda category, indicating that most initial nondiagnostic results were likely related to sampling adequacy rather than intrinsic lesion characteristics. These findings highlight the importance of optimizing the first FNA procedure in order to reduce the need for repeat sampling.

In contrast, repeat FNAs were documented in a smaller number of cases at Hospital for Tumors, UHC “Sisters of Charity”, and the outcomes should be interpreted cautiously due to the limited sample size. Nevertheless, the overall lower nondiagnostic rate observed at this center suggests that procedural factors, including operator specialization and the use of ROSE, may contribute to improved specimen adequacy.

Several limitations of this study should be acknowledged. First, the retrospective design limits control over potential confounding variables such as nodule size, cystic composition, ultrasound characteristics, and individual operator experience. Second, the study compared two institutional practice models in which operator specialization and ROSE implementation occurred simultaneously; therefore, the independent contribution of each factor cannot be separately determined. Third, repeat FNA follow‐up was limited to procedures performed within the 2025 study period, and additional repeat aspirations performed after this period were not captured. Fourth, although a standardized single‐pass approach was used at the Hospital for Tumors, data on the number of needle passes at UCCK were not systematically available. Therefore, the potential impact of the number of passes on specimen adequacy could not be directly assessed. Finally, histopathologic follow‐up was not systematically analyzed, preventing direct calculation of institution‐specific malignancy rates for Bethesda Category I.

Despite these limitations, the study provides valuable insight into institutional variability in thyroid FNA adequacy and highlights potential quality improvement strategies in cytopathology practice.

## 5 Conclusion

This study demonstrates a significantly lower rate of Bethesda Category I thyroid FNA results in a center where procedures were performed by interventional cytologists with routine implementation of rapid on‐site evaluation. The findings suggest that operator specialization and on‐site adequacy assessment may represent important quality indicators in thyroid cytopathology practice.

Given the reported 5%–20% risk of malignancy associated with nondiagnostic aspirates and the high diagnostic conversion rate observed after repeat FNA, strategies aimed at optimizing the initial sampling procedure may help reduce repeat procedures and improve diagnostic efficiency in the evaluation of thyroid nodules.

## Funding

The authors have nothing to report.

## Ethics Statement

This study protocol was reviewed and approved by the Ethics Committee of the Kosovo Doctors Chamber (approval no. 86/26) and by the Ethics Committee of UHC “Sisters of Charity”, Zagreb, Croatia (approval no. 251‐29‐11/3‐26‐05).

## Consent

The study was conducted on archived thyroid cytology specimens, and no direct patient contact was involved. The requirement for written informed consent was waived by both Ethics Committees.

## Conflicts of Interest

The authors declare no conflicts of interest.

## Data Availability

The data that support the findings of this study are available on request from the corresponding author. The data are not publicly available due to privacy or ethical restrictions.
